# Alveolar Abscess

**Published:** 1859-07

**Authors:** H. N. Wadsworth


### ARTICLE III.

### *Alveolar Abscess.* By H. N. Wadsworth, D. D. S.

Some two years ago I had a case of alveolar abscess, which it was of great moment to cure, as it was decided to insert two artificial crowns upon one root, which, when the root is a good one—can be done in such a manner as to be exceedingly comfortable and artistic, but as it is performed by most of the profession, it is not necessary to go over the method.

The root in question was the first left superior bicuspid; the second one adjoining having been extracted : my desire was to insert the two upon the power to be obtained by a gold pivot surrounded by wood, and inserted in the first bicuspid. This tooth had been operated upon some year or more previous, by the dentist who then had charge of my patient's teeth, (and who, I believe, was a skillful operator,) the pulp removed, and the pulp cavity and crown filled. Alveolar abscess had followed, a fistula had formed and the abscess would occasionally swell and break, giving more or less uneasiness.

Obliged to drill the nerve canal for my pivot, I determined to try the efficacy of creosote internally, and having succeeded in removing the gold from the fang, (a single one, having only a single nerve canal,) the application of creosote was commenced and persevered in until it became a settled fact in my mind that it was a waste of time and patience, and that some other course must be adopted.

Taking a square drill, a hole was then drilled (following the course indicated by a gold probe, which had first been carefully introduced through the channel formed by the fistulous opening, to the apex of the root, and which could be readily sounded) directly to the apex of the root; a gold tube inserted, through which was passed a gold wire, having its end bearded, a small bit of cotton twisted upon it and then rolled in finely pulverized nitrate of silver: in fact the instrument was a diminutive porti caustique, and the operation precisely that followed in cauterizing the male urethra in gonorrhœa and gleet; this plan was repeated three times, at intervals of three days, and after an apparent great improvement, the artificial work was put in situ and gave great satisfaction. Note the result! In less than a year we extracted the root, having decided our glorious theory, our nicely executed experiment—was a failure.

The fang showed evident marks of continued abscess! The application of a lance to the gum and thoroughly opening the fistula, then drilling through the alveoli and inserting a bit of cotton and its retention, applying creosote, &c., should be tried, remembering that the cotton should be retained at the bottom until healthy granulation commence below, otherwise the cotton will be expelled, the external orifice close, and the disease remain as before, but in this plan, too, I have failed, though a thorough trial has been repeatedly made.

In my practice thus far, chronic alveolar abscess has proved, like consumption to the medical man, incurable. Creosote and other agents have been freely, continuously, faithfully used, and my opinion is becoming fixed from the experiments thus far made in my own cases, that when well established in the alveolar cavity, it is incurable: it may be palliated, a tooth may be rendered comparatively comfortable and tolerably useful, but the secretion of pus goes on and never wholly ceases until the fang is removed.

Having formed this opinion, (which, however, is by no means to be understood to stop an earnest and continued effort to find a remedy,) it has been determined in my mind to try another plan in such cases as are propitious, and at the same time, when we can get our patient's consent. We all know that the removal of whatever cause prevents the escape of a suppurating pulp, be it gold, food or bone, will immediately give relief to incipient periostitis: we also know if this irritating pus is not allowed to discharge, it will bring on slight, at first, followed by violent, periosteal inflammation. That membrane goes rapidly into a condition of suppuration, and once in that condition, the opening of the nerve canal, the application of any and every remedy, blood letting included, serves only to palliate, it dont *cure;* that pus, like murder, "will out;" either it will raise the tooth from its socket and ooze at the free margin of the gum, or going to work at the weakest point of bone, it drills a hole, and a grand puff of the softer integuments shows at once the state our patient has arrived at, and he or she feels the relief afforded at this stage.

Suppose now we take our patient at that point in the disease when inflammation is evidently verging into suppuration, which all can know follows, if our topical applications, free opening of the channel of the nerves, leeches, *&c., &c.,* do not afford relief, and follows with certainty and dispatch. Suppose we run a drill down through the alveolus (first making an incision through the gum) to the apex of the root and let this terrible irritant have an opportunity to escape; so powerful a destroyer that it is capable of drilling a hole for itself: this sulphuretted hydrogen gas, this decomposed animal tissue, will readily and freely avail itself of this opportunity to escape and leave the surrounding parts in a measure uninjured; through this opening irritants may escape and remedies be applied.

Few patients will submit to this, but a few experiments will determine its practicability or its fallacy.

I have in no place seen the article arnica mentioned as applicable to periostitis; let me recommend it for that purpose, those who have not tried it will be gratified by applying it to the gums in that disease.

Some years ago, in the Dental News Letter, Philadelphia, it will be remembered, my mentioning a case where five branches were found running into the main pulp of a left superior cuspid, in which I failed in my operation in consequence of irritability remaining in these branches, (the main pulp was killed by mechanical means,) the question was then a very pointed one; had I killed this pulp with arsenicum, would not the operation have been successful? I now think it would! Several attempts to do as I did then have demonstrated the superiority of chemical means to mechanical for killing the pulp of a tooth, even a cuspid or incisor. Last summer another cuspid fell into my hands for a removal of the pulp, and with two distinct channels, out of which I removed two distinct pulps as well identified, even the canals, as those of a superior bicuspid.

WASHINGTON, *March* 22, 1859.

